# **Artificial intelligence in medical education - perception among medical students**

**DOI:** 10.1186/s12909-024-05760-0

**Published:** 2024-07-27

**Authors:** Preetha Jackson, Gayathri Ponath Sukumaran, Chikku Babu, M. Christa Tony, Deen Stephano Jack, V. R. Reshma, Dency Davis, Nisha Kurian, Anjum John

**Affiliations:** Pushpagiri Medical College, Tiruvalla, Kerala India

**Keywords:** Artificial intelligence, Medical curriculum, Healthcare, Medical ethics, Medical education

## Abstract

**Background:**

As Artificial Intelligence (AI) becomes pervasive in healthcare, including applications like robotic surgery and image analysis, the World Medical Association emphasises integrating AI education into medical curricula. This study evaluates medical students’ perceptions of ‘AI in medicine’, their preferences for AI training in education, and their grasp of AI’s ethical implications in healthcare.

**Materials & methods:**

A cross-sectional study was conducted among 325 medical students in Kerala using a pre-validated, semi structured questionnaire. The survey collected demographic data, any past educational experience about AI, participants’ self-evaluation of their knowledge and evaluated self-perceived understanding of applications of AI in medicine. Participants responded to twelve Likert-scale questions targeting perceptions and ethical aspects and their opinions on suggested topics on AI to be included in their curriculum.

**Results & discussion:**

AI was viewed as an assistive technology for reducing medical errors by 57.2% students and 54.2% believed AI could enhance medical decision accuracy. About 49% agreed that AI could potentially improve accessibility to healthcare. Concerns about AI replacing physicians were reported by 37.6% and 69.2% feared a reduction in the humanistic aspect of medicine. Students were worried about challenges to trust (52.9%), patient-physician relationships (54.5%) and breach of professional confidentiality (53.5%). Only 3.7% felttotally competent in informing patients about features and risks associated with AI applications. Strong demand for structured AI training was expressed, particularly on reducing medical errors (76.9%) and ethical issues (79.4%).

**Conclusion:**

This study highlights medical students’ demand for structured AI training in undergraduate curricula, emphasising its importance in addressing evolving healthcare needs and ethical considerations. Despite widespread ethical concerns, the majority perceive AI as an assistive technology in healthcare. These findings provide valuable insights for curriculum development and defining learning outcomes in AI education for medical students.

**Supplementary Information:**

The online version contains supplementary material available at 10.1186/s12909-024-05760-0.

## Introduction

The concept of Artificial Intelligence (AI) dates back to the 1950s when Alan Turing, often referred to as the father of computer science, proposed the question, “Can machines think”? Interestingly, he designed the now famous ‘Turing Test’ where humans were to identify the responder of a question as human or machine [1]. Subsequently in 1956 John McCarthy coined the term “Artificial Intelligence” [2] and the next decade saw the birth of the first ever artificial neural network which was “the first machine which is capable of having an original idea” [3]. Thus progressed the growth of this once unimaginable phenomenon. In this 21st century, most people are familiar with the term AI because of Siri (Intelligent Virtual Assistant) [4], Open AI’s ChatGPT (language model based chatbot) [5], traffic prediction by Google Maps or Uber [6] or customer service bots (AI powered assistants) [4] that intelligently provide suggestions.

There is no universally accepted definition for AI, but it can be simply defined as “the ability of machines to mimic intelligent human behaviour, including problem solving and learning” [7]. Specific applications of AI include expert systems, natural language processing, speech recognition, machine vision, and many more, applying which AI has exhibited qualities similar to or even above those of humans [8].

The use of AI and related technologies is becoming increasingly prevalent in all aspects of human life and beginning to influence the field of healthcare too [9]. AI technologies have already developed algorithms to analyse a variety of health data, including clinical, behavioural, environmental, and drug information using data from both patients as well as biomedical literature [10]. Convoluted Neural Networks, designed to automatically and adaptively learn spatial hierarchies of features, can be successfully used to develop diabetic retinopathy screening [11], skin lesion classification [12], lymph node metastasis detection [13], and detection of an abnormality in a radiograph [14].

Artificial Intelligence can help patients understand their symptoms, influence health seeking behaviour, and thereby improve their quality of life [15]. AI assistants have even suggested medicines for cancer patients with equal or better efficiency than human experts [16]. With a capable AI assistant, it is possible to sift through and analyse multitudes of data in a matter of seconds and make conclusions, thus exponentially increasing its applications in biomedical research. AI promises future influences in healthcare in terms of AI assisted robotic surgery, virtual nursing assistants, and image analysis. Simply put, AI can help patients and healthcare providers in diagnosing a disease, assessing risk of disease, estimating treatment success, managing complications, and supporting patients [17].

Though AI has limitless potential, it has certain vulnerabilities and weaknesses. The quality and relevance of the input data can affect the accuracy of a deep learning diagnostic AI.The kind of funding that is required to construct the machinery and develop an intelligence is not easily accessible in the field of medicine, not to mention the constraints of machine ethics and confidentiality. However, being familiar with the concepts, applications and advantages of AI is definitely beneficial and therefore advisable, especially in the field of medical education and policy making [17, 18].

The World Medical Association advocates for a change in medical curricula and educational opportunities for patients, physicians, medical students, health administrators, and other health care professionals to foster a better understanding of the numerous aspects of the healthcare AI, both positive and negative [19]. Additionally, in 2019, the Standing Committee of European Doctors stressed the need to use AI systems in basic and continuing medical education [20]. They recommended the need for AI systems to be integrated into medical education, residency training, and continuing medical education courses to increase awareness of the proper use of AI. In this context, there is an emerging need for developing curricula specifically designed to train future physicians on AI.

To develop an effective AI curriculum, we need to understand how today’s medical students perceive AI in medicine, and their comprehension of AI’s ethical dimension as well. However, the available need assessment studies in an Indian setting are barely enough. Grunhut et al. had recommended in 2021 that national surveys need to be carried out among medical students on the attitude and expectations of learning AI in medical colleges for developing a curriculum [21]. Similar unbiased probability based, large scale surveys would identify the realistic goals physicians will be asked to meet, the expectations that will be put on them, and the resources and knowledge they would need to meet these goals. Also, current literature falls short of a comprehensive needs assessment which is important for curriculum development and defining learning outcomes. Hence in this study we aimed to assess the perceptions on ‘AI in medicine’ among Indian medical students, to assess the proportion of medical students who are in favour of structured training on AI applications during their undergraduate course, and also to assess their perceptions on AI’s ethical dimensions.

## Methods

Recruitment: A cross-sectional study was conducted among the undergraduate medical students of Pushpagiri Institute of Medical Sciences and Research Centre during the period of June – August 2023. An introductory discussion on the purpose and importance of this study was conducted with each batch of students from first year to house surgeons following which the link to the Google-form containing the consent and questionnaire was shared in the batch Whatsapp groups.

There were a total of 500 medical students in the Institute from 1st year MBBS to the medical students undergoing their internship. The Google form was open for 3 months, with reminder messages sent at intervals of one month. Participation was voluntary (informed consent was obtained through the first section of the Google form)due to which no randomisation could be ensured, implying that some selection bias can be expected.

Participants who did not consent or submitted incomplete questionnaires were excluded from the study. An online survey using Google forms was conducted using a validated semi structured questionnaire which had 3 sections. The questions were adopted from a Turkish study by Civaner et al. [22]. Since the questionnaire was originally drafted in English, there was no need for translation into a comprehensible language. The first section dealt with demographic details (age, gender and year of study), any past educational experience about AI (had attended training or seminars) and participants’ self-evaluation of their knowledge of AI. The second section consisted of 12 five point Likert questions on medical students’ perceptions of AI including five questions on ethical aspects as well, which were expressed in the form of agreement or disagreement. The last section was about their opinions on selected topics on AI - whether they should be included in their medical curriculum or not. A pilot study was undertaken by administering the questionnaire to a group of 20 medical students who were then posted in the Department of Community Medicine.

Statistical Analysis: Responses on medical students’ perception on the possible influences of AI were graded using Likert scale ranging from 0 (totally disagree) to 4 (totally agree). Data was entered into Microsoft Excel and analysed using Statistical Package for Social Sciences 25.0. Age of the participants is expressed as mean with standard deviation and categorical variables such as opinions, perceptions and year of study are expressed as frequencies and percentages.

## Results

Out of 500 medical students in the institution, 327 students participated in the survey. After excluding the incomplete questionnaires, data of 325 participants were analysed. Therefore the response rate amounts to 65%.

The mean (SD) age of the participants was 21.4 (1.9) years, (ranging from 18 to 25 years) with 76% (248/325) females.

### AI in medicine- prior knowledge and self-evaluation

Majority of students (91.4%)(297/325) stated that they had not received any training on AI in their medical curriculum, while the others mentioned that they had attended events like seminars and presentations on AI. Almost 52%(169/325) students have heard about AI but possess no knowledge of it. One third of the participants (106/325) self-reported to have ‘partial knowledge’ on AI while none of them reported to be ‘very knowledgeable.’

Of all the participants, only 37.2% (121/325) did not agree with the opinion that AI could replace physicians; instead, the majority thought that it could be an assistant or a tool that would help them. About 37.6% (122/325) of participants agreed that the use of AI would reduce the need for physicians and thus result in loss of jobs. More than half of the participants (173/325) agreed that they would become better physicians with the widespread use of AI applications. Almost 35% (114/325) stated that their choice of specialization would be influenced by how AI was used in that field. Only 26.8% (87/325) of participants totally or mostly agreed that they felt competent enough to give information on AI to patients. More than half of the participants (166/325) were unsure of protecting patient confidentiality while using AI.

### Perceptions on the possible influences of AI in medicine

**Fig. 1:**
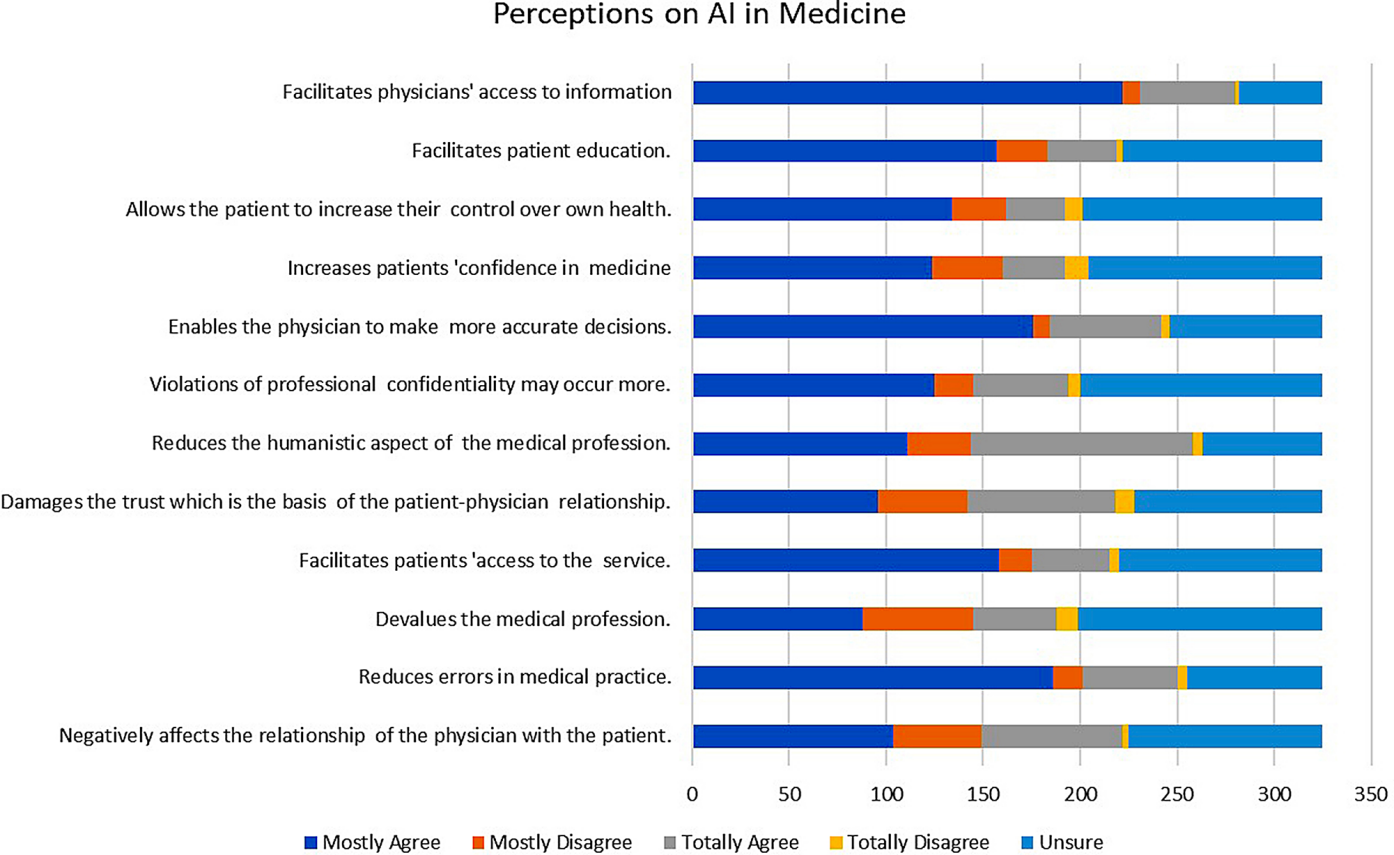
Frequency distribution of perceptions of medical students on AI in medicine

Regarding student perceptions on the possible influences of AI in medicine (Fig. 1), the highest agreement (72.3%) was observed on the item ‘reduces error in medical practice’ (235/325) while the lowest agreement (40.3%) was on ‘devalues the medical profession’ (131/325). Students were mostly in favour of applying AI in medicine because they felt it would enable them to make more accurate decisions (72%, (234/325) and would facilitate patients’ access to healthcare (60.9%, 198/325). There were 59.4% (193/325) of participants who agreed that AI would facilitate patient education and 50.5% (164/325) who agreed that AI would allow the patient to increase their control over their own health.

### Need for training on AI in medical curriculum

**Fig. 2:**
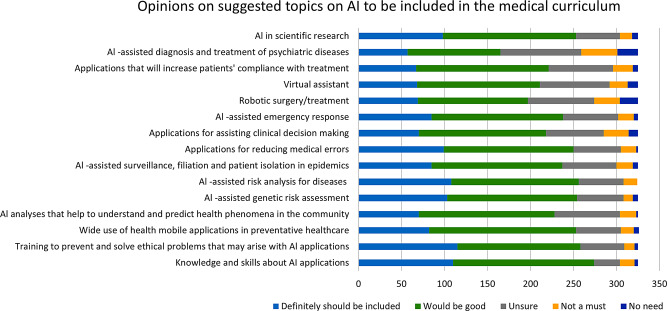
Frequency distribution of opinions of medical students as to whether the suggested topics should be included in their medical curriculum

Almost three-fourths of the participants were in favour of structured training on AI applications that should be given during medical education (74.8%, 243/325). The participants thought that it was important to be trained on various topics related to AI in medicine as depicted in Fig. 2. The most frequent topics that they perceived necessary in this domain were knowledge and skills about AI applications (84.3%274/325), training to prevent and solve ethical problems that may arise with AI applications (79.4%258/325), and AI assisted risk analysis for diseases (78.1%254/325).

### Ethical concerns regarding AI in medicine (Table 1)

On the topic of disadvantages and risks of using AI in medicine 69.2% (225/325) agreed that AI would reduce the humanistic aspect of the medical profession, 54.5% (177/325) agreed that it could negatively affect the patient-physician relationship, 52.9% (173/325) were concerned that using AI assisted applications can damage trust in patients while 53.5% (174/325) thought that AI could possibly cause violations of professional confidentiality.

**Table 1:** Opinions of medical students on ethical considerations of including AI in medicine

Concerns	Totally agree	Mostly agree	Unsure	Mostly disagree	Totally disagree
Negatively affects patient-physician relationship	73	104	100	45	3
Devalues the medical profession	43	88	126	57	11
Damages trust	76	96	97	46	10
Reduces the humanistic aspect of the medical profession	114	111	62	33	5
Violations of professional confidentiality	49	125	125	20	16

### Sub group analysis

Perceptions about being a better doctor with the use of AI applications, being competent enough to inform patients about features & risks of AI applications and the perception about the use of AI in medicine causing a reduction in job opportunities were the ones which showed significant association with the baseline variables like gender, year of study and having prior exposure to course of AI applications as shown in Table 2.

**Table 2:** Association between gender, year of study and previous training in AI with selected statements for perceptions

		Gender		Year of study		Have you received training in AI at your medical school or elsewhere?	
		Male	Female	Pre-clinical	Clinical	Yes	No
How much do you agree with the statement “I think I will be a better doctor with the widespread use of AI applications.”	Agree	46 (59%)	127 (51.4%)	93 (56.4%)	80 (50%)	21 (75%)	152 (51.2%)
	Disagree	12 (15.4%)	18 (7.3%)	13 (7.9%)	17 (10.6%)	2 (7.1%)	28 (9.4%)
	Unsure	20 (25.6%)	102 (41.3%)	59 (35.8%)	63 (39.4%)	5 (17.9%)	117 (39.4%)
p value*		0.013**		0.457		0.049**	
How much do you agree with the statement “Currently, I feel competent enough to inform patients about the features and risks of AI applications.”	Agree	33 (42.3%)	54 (21.9%)	54 (32.7%)	33 (20.6%)	15 (53.6%)	72 (24.2%)
	Disagree	15 (19.2%)	66 (26.7%)	31 (18.8%)	50 (31.3%)	3 (10.7%)	78 (26.3%)
	Unsure	30 (38.5%)	127 (51.4%)	80 (48.5%)	77 (48.1%)	10 (35.7%)	147 (49.5%)
p value		0.002**		0.009**		0.003**	
How much do you agree with the statement “The use of AI in medicine reduces the need for physicians and thus employment opportunities”	Agree	29 (37.2%)	93 (37.7%)	63 (38.2%)	59 (36.9%)	9 (32.1%)	113 (38%)
	Disagree	35 (44.9%)	86 (34.8%)	70 (42.4%)	51 (31.9%)	13 (46.4%)	108 (36.4%)
	Unsure	14 (17.9%)	68 (27.5%)	32 (19.4%)	50 (31.3%)	6 (21.4%)	76 (25.6%)
Total		78	247	165	160	28	297
p value		0.152		0.03**		0.574	

* Pearson Chi Square Test was done

**significant at 0.05

## Discussion

Although there has been extensive research on the utilisation of AI in medical education the perceptions of medical professionals, and their dilemmas regarding its integration into their daily practice remains relatively underexplored. This research is focused on the perception of medical students about the use of Artificial Intelligence in medicine and its ethical aspects, which reflects their confusions and concerns regarding the situation.

The mean age of the medical students studied was around 21 years and the majority of students were females. Most participants in our study (53.3%) agreed that AI could not replace the presence of a physician but could help them in their work. This is in accordance with the 2021 study conducted by Bisdas S et al. on medical students from 63 countries that AI could work as a “partner” rather than as a “competitor” in their medical practice. A third of our participants (37.6%) felt that the use of AI would reduce the need for physicians and would result in a loss of job opportunities for them. This is a different finding than the study published by D Pinto Dos Santos in European Radiology in 2019 where a majority of participants (83%) felt that human radiologists would not be replaced by robots or computers [23]. In fact, there are many studies which argue that rather than physicians becoming redundant because of AI, they would change their practice and become “managers” rather than “custodians of information” [24, 25].

More than half the respondents in our study (53.3%) agreed that they would become better physicians with the widespread use of AI applications. This is in concurrence with a recently published Western Australian study among medical students which showed about 75% of the participants agreeing that AI would improve their practice [26]. Respondents from other studies felt that currently available AI systems would actually complement physicians’ decision-making skills by synthesising large amounts of medical literature in order to produce the most up-to-date medical protocols and evidence [27–30]. Similarly, studies show that AI systems actually work by complementing the practice of medicine, rather than competing with human minds. After all, human minds have designed artificial intelligence. Furthermore, the study by Paranjape et al. comments that physicians will be able to focus on providing patients with the humanistic care considering the biopsychosocial model of disease as the technicalities can be handled by the AI supported technologies to a greater extent [28].

A third of the participants (35.1%) in our research stated that their choice of specialisation would be influenced by how AI was used in that field. Much has been written about how AI might replace specialists in the fields of radiology and pathology as perceived by medical doctors and students. These are specialisations that use computers and digital algorithms more when compared to other medical specialties. A Canadian study published in 2019 by Bo Gong et al. found that 67% of the respondents felt that AI would “reduce the demand” for radiologists. Many of the medical students interviewed in this study said that the anxiety they felt about being “displaced” by AI technologies in radiology would discourage them from considering the field for specialisation [14, 31–33]. In fact, a paper published by Yurdasik et al. in 2021 had respondents encouraging practitioners to move away from specialisations that used AI [34]. However, there were other studies that reported results encouraging radiologists to get exposed to AI technologies so as to lower the rates of “imaging related medical errors” and “lessening time spent in reading films,” resulting in more time spent with patients. German medical students have shown a positive attitude towards AI and have reported “not being afraid of being replaced by AI” should they choose radiology as their specialisation [23]. Attitude towards the choice of specialisation being influenced by AI depended on where the person was viewing the problem from- as a student or as a specialist and also from the degree of familiarity they had with AI applications.

The majority of the students (91.4%) stated that they had not received any training on AI in medicine. The American Medical Association meeting of 2018 on Augmented Intelligence advocated for the training of physicians so that they could understand algorithms and work effectively with AI systems to make the best clinical care decisions for their patients [35]. Despite this, Paranjape et al. reported that training on the backend of electronic health record systems like, the quality of the data obtained, impact of computer use in front of patients, patient physician relationships etc. have not been addressed through medical education. If used with adequate training and understanding, AI will free up physicians’ time/ optimise a physician’s work hours, so that they can care and communicate with patients in the free time thus obtained. The findings of the research published by Jha et al. in the year 2022 agrees with this observation regarding inadequate coverage of AI and machine learning in medical curricula [36]. This deficiency leaves medical students underprepared to navigate the integration of AI technologies into their future practice. A significant percentage (37.6%) of respondents expressed concerns about job displacements due to AI, echoing sentiments observed in previous research. The concerns on AI induced loss of jobs particularly in fields like radiology and pathology, accentuate the importance of addressing misconceptions and fostering a meticulous understanding of AI’s role in healthcare. Jha et al’s study also highlights the importance of integrating soft skills, such as compassion and empathy, alongside AI education. Medical students must be equipped not only with technical AI competencies but also with the interpersonal skills necessary for holistic patient care. Collaborative efforts are needed to develop curricula that balance AI education with the cultivation of humanistic values, ensuring that future healthcare professionals can effectively navigate the intersection of technology and patient-centred care.

A major proportion of students in the study conducted by Sharma et al. demonstrated only a limited understanding of AI’s applications in medicine, primarily attributed to a lack of formal education, awareness, and interest. Interestingly, while a substantial portion (26.3%) of respondents demonstrated familiarity with AI, the majority (53.6%) exhibited only a superficial understanding of its applications in medicine [37]. This gap in knowledge highlights the need for enhanced educational initiatives to provide comprehensive insights into the potential of AI in healthcare delivery and patient outcomes. Concerns about the overreliance (49.2%) on AI and perceived lack of empathy (43.7%) were highlighted by a considerable proportion of students. These concerns underscore the importance of fostering a balanced approach to AI adoption in medical practice and education, ensuring that students are equipped to navigate the ethical challenges associated with AI integration.

Medical curriculum does not address mathematical concepts (to understand algorithms), the fundamentals of AI like data science, or the ethical and legal issues that can come up with the use of AI [27]. Only 26.8% of participants felt partially or completely competent to give information on AI to patients. Unless physicians have a foundational understanding of AI, or the methods to critically appraise AI, they will be at a loss when called to train medical students on the use of AI tools that assist in medical decision making. Consequently, medical students will be deficient in AI skills. Liaw et al. advocate for Quintuple Competencies for the use of AI in primary health care, one of which is the need to understand how to communicate with patients regarding the why and how of the use of AI tools, privacy and confidentiality questions that patients may raise during patient physician interactions, and understand the emotional, trust or patient satisfaction issues that may arise because of use of AI in health care [38].

More than half of the participants (51.1%) are unsure of being able to protect professional confidentiality of patients during the use of AI technologies. Direct providers of health care need to be aware of what precautions to take when sharing data with third parties who are not the direct care providers to the patients [16]. Artificial intelligence algorithms are derived from large data sets from human participants, and they may use data differently at different points in time. In such cases, patients can lose control of information they had consented to share especially where the impact on their privacy have not been adequately addressed [39]. However much regulations might be made to protect patient confidentiality and privacy of data, they might always fall behind AI advances, which means the human brain has to work consistently to remain ahead of the artificial intelligence it created. Guidelines set forth by reputable organisations such as the European Union’s “Guidelines for Trustworthy AI“ [40] and the World Health Organization’s “Ethics and Governance of Artificial Intelligence for Health” address critical ethical concerns in AI [41]. These core principles can be integrated into medical education to cultivate ethical awareness among medical students.

The perceptions of medical students on the possible influences of AI in medicine were evaluated through the questionnaire. The highest agreement was found on the question, whether they thought the use of AI ‘reduces error in medical practice’ (72.3%) while the lowest agreement was on the question AI ‘devalues the medical profession’ (40.3%).Students were mostly in favour of the use of AI in medicine because they felt that it would enable them as physicians to make more accurate decisions (72%) and facilitate patients’ access to healthcare (60.9%). Research by Topol et al. and Sharique et al. have shown that AI technologies can help reduce medical errors by improving data flow patterns and improving diagnostic accuracy [39, 42]. The study from Western Australian students mentioned above [26] showed 74.4% of the participants agreeing that the use of AI would improve practice of medicine in general. It is encouraging to find that medical students in this research showed low agreement when asked if AI would devalue the medical profession and agreed that the use of AI would reduce medical errors caused inadvertently. It should also be noted that some research has shown that the inappropriate use of AI itself can introduce errors in medical practice [43].

On “disadvantages and risks of AI in medicine”, 69.2% of the students agreed that AI would reduce the humanistic aspect of the medical profession, 54.5% agreed that it can negatively affect the patient-physician relationship, 52.9% were concerned that using AI assisted applications could damage the trust patients placed on physicians, 59.4% agreed that AI would facilitate patient education, and 50.5% agreed that AI would allow the patient to increase their control over their own health. Hadithy et al. (2023) found that students believed AI technology was advantageous for improving overall health by personalising health care through analysing patient information [44].

Medical education in the 21st century is swiftly transitioning from the conventional approach of observing patients objectively from a distance and holding the belief that compassion is an innate skill to a contemporary paradigm. The new model emphasises the development of competencies such as doctor-patient relationships, communication skills, and professionalism. In modern medicine, AI is being viewed as an additional barrier between a patient and his physician. Machines have many advantages over humans as rightly observed by Wartman especially in view of not being affected by many of the human frailties like fatigue, information overload, inability to retain material beyond a limit etc. [24]. Scepticism over the use of AI in medical practice often stems from the lack of knowledge in this domain. Medical students, in many studies, opined that classes on artificial intelligence need to be included in syllabus, but only very few medical schools have included these in their medical curricula. Practising with compassion and empathy must be a learnt and cultivated skill along with artificial intelligence. The two should go together, taught in tandem throughout the medical course. Studies such as this have highlighted that students are open to being taught but are deficient in the skills and knowledge. There is a gap here that needs to be addressed. Man, and machine have to work as partners so as to improve the health of the people.

## Limitations

Though this research was one of the first conducted in the state of Kerala and covered about 65% of medical students of the institution, which is more than other similar surveys conducted, there are a few limitations that have been identified. As an online survey method using Google Forms was implied for data collection, the voluntary nature of the participation from only those who were interested, might have introduced a self-selection bias and a non-response bias in this research. As this study only includes the responses from the medical students of one institution, it might not have captured a wide variety of responses. Hence the generalizability of the study may be limited. The questionnaire did not delve deep into how AI terms are understood, or how proficient students were with AI and so might have missed more relevant AI terms and concepts that students might be unfamiliar with. Most data collected in this study were quantitative so we might not have captured the depth of the students’ understanding or perceptions about AI. As many of the students had no exposure to computer science or had not attended AI classes, their perceptions might have been influenced by lack of exposure. Thus, the study might not have captured the views of those who had a more informed background on the subject.

Future studies are recommended to replicate and validate the findings in larger and more diverse populations to understand regional variations in knowledge, attitude, and perceptions among medical students. This study tool (questionnaire) was adopted from a parent study by Civaner M M [10], but the last question on the need for any other topic to be included was not met with enthusiasm.

## Conclusion

This exploration into the perceptions of medical students regarding the integration of Artificial Intelligence (AI) into medical education reveals a nuanced landscape. The majority of participants in this study recognize the collaborative potential of AI, viewing it not as a replacement for physicians but as a valuable ally in healthcare. Interestingly, concerns on job displacement coexist with the optimism about improved decision-making and enhanced medical practice. The knowledge deficit in this context can extend an incompetence in communicating AI related information to patients, highlighting the urgent need for a holistic approach to medical education. The findings complement the perceived need of a proactive approach in preparing medical students for a future where AI plays a pivotal role in healthcare, ensuring that they not only embrace technological advancements but also uphold the humanistic values inherent to the practice of medicine.

### Electronic supplementary material

Below is the link to the electronic supplementary material.

Supplementary Material 1

Supplementary Material 2

## Data Availability

Data is provided as supplementary information files.

## Abbreviations

AI: Artificial Intelligence
IIT: Indian Institute of Technology
