# Supplementary material for: Artificial intelligence in medical education - perception among medical students
Source: BMC Med Educ. 2024 Jul 27;24:804. doi: 10.1186/s12909-024-05760-0 (PMC11283685; doi:10.1186/s12909-024-05760-0)
Supplement: Supplementary file 1 — Supplementary Material 1 [file 12909_2024_5760_MOESM1_ESM.docx]

Annexures

1. Informed Consent

2. Questionnaire

**1. Study Participant Consent Form**

I, hereby agree voluntarily to include myself in the project “ARTIFICIAL INTELLIGENCE IN MEDICAL EDUCATION - PERCEPTION AMONG MEDICAL STUDENTS” conducted by Dr. Preetha Jackson. I understand that I need not take any financial burden for the study. I am also aware that by participating in this study I will not be exposed to any physical or mental harm. I am fully aware that my identity will not be revealed at any stage of the study. I give my full consent to participate in the study for the purpose of this thesis.

Signature of the Subject -

Signature of the Investigator-

Date:

**2. Questionnaire**

1. Age ………………………..

2. Gender F ⃝ M ⃝ Others

3. Year 1 ⃝ 2 ⃝ 3 ⃝ 4 ⃝ 5 ⃝

4. When choosing a field of specialization, is your choice affected by how artificial intelligence is used in that field?

a)Yes b)No c)Not sure

5. Have you received training in AI at your medical school or elsewhere? (more than one can be marked)

a) No; I received no training.

b) I took it as an elective course.

c) I attended events such as seminars and presentations.

d) It was included as a subject in a required course.

e) I attended an online training.

f) Other: ……………………….....

6. How would you describe your level of knowledge about AI applications in medicine?

a) I have no knowledge

b) I have heard about it but possess no knowledge

c) I have partial knowledge

d) I am quite knowledgeable

e) I am very knowledgeable

7. Can you evaluate the reliability of a diagnostic application using AI?

a) I can definitely evaluate it.

b) I think I can mostly evaluate.

c) I am not sure.

d) I generally think that I cannot evaluate.

e) I absolutely cannot evaluate it.

8. How much do you agree with the statement “The use of AI in medicine reduces the need for physicians and thus employment opportunities”?

a) Totally agree

b) Mostly agree

c) Unsure

d) Mostly disagree

e) Totally disagree

9. How much do you agree with the statement “It cannot replace the physician; but it can help him.”

a) Totally agree

b) Mostly agree

c) Unsure

d) Mostly disagree

e) Totally disagree

10. How much do you agree with the statement “I think I will be a better doctor with the widespread use of AI applications.”

a) Totally agree

b) Mostly agree

c) Unsure

d) Mostly disagree

e) Totally disagree

11. How much do you agree with the statement “Currently, I feel competent enough to inform patients about the features and risks of AI applications.”

a) Totally agree

b) Mostly agree

c) Unsure

d) Mostly disagree

e) Totally disagree

12. How much do you agree with the statement “I think I can protect professional confidentiality when using AI applications.”

a) Totally agree

b) Mostly agree

c) Unsure

d) Mostly disagree

e) Totally disagree

13. Are you in favour of structured training in AI applications during medical education?

a) Yes

b) No

14. Please choose to which degree you agree with the statements below on the possible effects of AI applications in medicine in the table below:

| Perceptions statements | Totally agree | Mostly agree | Unsure | Mostly disagree | Totally disagree |
| --- | --- | --- | --- | --- | --- |
| Negatively affects the relationship of the physician with the patient. |  |  |  |  |  |
| Reduces errors in medical practice. |  |  |  |  |  |
| Devalues the medical profession. |  |  |  |  |  |
| Facilitates patients 'access to the service. |  |  |  |  |  |
| Damages the trust which is the basis of the patient-physician relationship. |  |  |  |  |  |
| Reduces the humanistic aspect of the medical profession. |  |  |  |  |  |
| Facilitates physicians 'access to information. |  |  |  |  |  |
| Violations of professional confidentiality may occur more. |  |  |  |  |  |
| Enables the physician to make more accurate decisions. |  |  |  |  |  |
| Increases patients 'confidence in medicine. |  |  |  |  |  |
| Allows the patient to increase their control over own health. |  |  |  |  |  |
| Facilitates patient education. |  |  |  |  |  |

15. Please choose to which degree you think the given titles should be included in the medical curriculum?

| Title | Definitely should be included | Would be good | Unsure | Not a must | No need |
| --- | --- | --- | --- | --- | --- |
| Knowledge and skills about AI applications |  |  |  |  |  |
| Training to prevent and solve ethical problems that may arise with Al applications |  |  |  |  |  |
| Wide use of health mobile applications in preventative healthcare |  |  |  |  |  |
| Al analyses that help to understand and predict health phenomena in the community |  |  |  |  |  |
| Al-assisted genetic risk assessment |  |  |  |  |  |
| Al-assisted risk analysis for diseases |  |  |  |  |  |
| Al-assisted surveillance, filiation and patient isolation in epidemics |  |  |  |  |  |
| Applications for reducing medical errors |  |  |  |  |  |
| Applications for assisting clinical decision making |  |  |  |  |  |
| Al-assisted emergency response |  |  |  |  |  |
| Robotic surgery/treatment |  |  |  |  |  |
| Virtual assistant |  |  |  |  |  |
| Applications that will increase patients' compliance with treatment |  |  |  |  |  |
| Al-assisted diagnosis and treatment of psychiatric diseases |  |  |  |  |  |
| Al in scientific research |  |  |  |  |  |
